# Melatonin Prevents Osteoarthritis-Induced Cartilage Degradation via Targeting MicroRNA-140

**DOI:** 10.1155/2019/9705929

**Published:** 2019-12-14

**Authors:** Yijian Zhang, Jun Lin, Xinfeng Zhou, Xi Chen, Angela Carley Chen, Bin Pi, Guoqing Pan, Ming Pei, Huilin Yang, Tao Liu, Fan He

**Affiliations:** ^1^Department of Orthopaedics, The First Affiliated Hospital of Soochow University, Soochow University, Suzhou 215006, China; ^2^Orthopaedic Institute, Medical College, Soochow University, Suzhou 215007, China; ^3^School of Public Health and Health Systems, University of Waterloo, Waterloo, Ontario, Canada N2L 3G1; ^4^Institute for Advanced Materials, School of Materials Science and Engineering, Jiangsu University, Zhenjiang 212013, China; ^5^Stem Cell and Tissue Engineering Laboratory, Department of Orthopaedics and Division of Exercise Physiology, West Virginia University, Morgantown, WV 26506, USA

## Abstract

Osteoarthritis (OA) is characterized by the progressive destruction of articular cartilage, which is involved in the imbalance between extracellular matrix (ECM) synthesis and degradation. MicroRNA-140-5p (miR-140) is specifically expressed in cartilage and plays an important role in OA-induced matrix degradation. The aim of this study was to investigate (1) whether intra-articular injection of melatonin could ameliorate surgically induced OA in mice and (2) whether melatonin could regulate matrix-degrading enzymes at the posttranscriptional level by targeting miR-140. In an *in vitro* OA environment induced by interleukin-1 beta (IL-1*β*), melatonin treatment improved cell proliferation of human chondrocytes, promoted the expression of cartilage ECM proteins (e.g., type II collagen and aggrecan), and inhibited the levels of IL-1*β*-induced proteinases, such as matrix metalloproteinase 9 (MMP9), MMP13, ADAMTS4 (a disintegrin and metalloproteinase with thrombospondin motifs 4), and ADAMTS5. Both the microarray and polymerase chain reaction (PCR) experiments revealed that miR-140 was a melatonin-responsive microRNA and melatonin upregulated miR-140 expression, which was suppressed by IL-1*β* stimulation. *In vivo* experiments demonstrated that intra-articular injection of melatonin prevented disruptions of cartilage matrix homeostasis and successfully alleviated the progression of surgery-induced OA in mice. Transfection of miR-140 antagomir completely counteracted the antiarthritic effects of melatonin by promoting matrix destruction. Our findings demonstrate that melatonin protects the articular cartilage from OA-induced degradation by targeting miR-140, and intra-articular administration of melatonin may benefit patients suffering from OA.

## 1. Introduction

Osteoarthritis (OA) is a chronic degenerative joint disease, which is primarily characterized by progressive destruction of articular cartilage. In the late stages of this pathology, OA-induced joint dysfunction is considered to be a leading cause of disability in elderly people, which creates a huge economic burden on society. Currently, no therapy has been shown to effectively halt the progression of OA, and the only clinical treatment for patients with late-stage OA is prosthetic implants [1]. Although the pathogenesis of OA is not yet fully understood, a key factor is the imbalance between extracellular matrix (ECM) synthesis and degradation in cartilage [2]. Chondrocytes, the only cell type in articular cartilage, produce the structural components of the cartilage ECM, specifically, type II collagen (Collagen II) and the proteoglycan aggrecan. However, during OA pathogenesis, chondrocytes become metabolically active to produce matrix-degrading enzymes; these enzymes include members of the matrix metalloproteinase (MMP) and ADAMTS (a disintegrin metalloproteinase with thrombospondin motifs) families, which are responsible for the degradation of Collagen II and aggrecan, respectively [3]. Among these enzymes, MMP13 (collagenase-3) can cleave collagen, aggrecan, and fibronectin and has the highest activity toward Collagen II [4]. ADAMTS4 (aggrecanase-1) and ADAMTS5 (aggrecanase-2) have also been shown to play important roles in OA development [5].

Several recent studies have demonstrated that the gene expression of matrix-degrading enzymes is tightly regulated by microRNAs (miRNAs) at the posttranscriptional level [6]. miRNAs are a class of small (19-24 nucleotides in length), noncoding RNAs that can regulate gene expression by binding to specific sequences in messenger RNAs (mRNAs), resulting in either degradation of the target mRNAs or repression of their translation [7]. The differential expression of miRNAs between normal and OA cartilage has been identified recently using miRNA microarrays [8]. Among them, microRNA-140-5p (miR-140), which is expressed specifically in cartilage, has been considered a key factor in chondrocyte differentiation and OA-induced matrix degradation. Deficiency of miR-140 in mice resulted in age-related OA-like changes in cartilage, such as the loss of proteoglycan and fibrillation of articular cartilage [9]. Additionally, miR-140 is dysregulated by increased levels of proinflammatory cytokines in OA cartilage, such as interleukin-1 beta (IL-1*β*) and tumor necrosis factor alpha (TNF-*α*). The expression of miR-140 rapidly decreased in IL-1*β*-stimulated chondrocytes, suggesting that miR-140 might be implicated in OA cartilage dyshomeostasis [10]. Therefore, miRNA therapeutics, especially regarding strategies targeting miR-140, is a promising treatment option for OA patients.

Melatonin, a hormone secreted mainly from the pineal gland, has been shown to exert beneficial effects on bone diseases, such as osteoporosis, osteopenia, and periodontal disease [11]. Previous studies from our laboratory and other groups have demonstrated that melatonin can enhance the chondrogenic differentiation of mesenchymal stem cells (MSCs), even in a proinflammatory cytokine-induced environment [12, 13]. Recently, a study suggested that the underlying mechanisms involved in melatonin-improved chondrogenesis was through the upregulation of miRNAs, such as miR-526b-3p and miR-590-5p, which activated the Smad signaling pathway by targeting SMAD7, a negative regulator [14]. However, it is unknown whether melatonin can protect articular cartilage from OA-induced matrix degradation, and few investigations have revealed the role of melatonin in regulating miR-140 during OA pathogenesis.

In this study, we investigated the protective effects of melatonin on OA-impaired articular cartilage. To establish an *in vitro* OA environment, IL-1*β* was supplemented during the cell culture of human articular chondrocytes, and the effects of melatonin on ECM synthesis and degradation were evaluated. In *in vivo* experiments, destabilization of the medial meniscus (DMM) surgery was performed to establish an OA mouse model, followed by intra-articular injection of melatonin for up to four weeks. To further investigate the underlying mechanisms, globe miRNA expression analysis was performed and the role of miR-140 in melatonin-mediated antiosteoarthritic effects was investigated.

## 2. Materials and Methods

### 2.1. Human Cartilage Sampling and Chondrocyte Preparation

The study protocol for using discarded human cartilage samples was reviewed and approved by the Ethics Committee of the First Affiliated Hospital of Soochow University. Cartilage samples were obtained from six OA patients (3 males and 3 females, age 60.4 ± 11.6) who underwent total joint replacement.

Articular cartilages from the femoral condyle and tibial plateau were minced into pieces and sequentially digested with 2 mg/mL type II collagenase (Thermo Fisher Scientific, Waltham, MA, USA) at 37°C overnight. Undigested tissue remnants were removed using a 100 *μ*m nylon mesh (BD Biosciences, San Jose, CA, USA). The isolated chondrocytes were seeded into 175 cm^2^ culture flasks (Costar, Tewksbury, MA, USA) and cultured in Dulbecco's modified Eagle's medium: nutrient mixture F-12 (DMEM/F-12) containing 10% fetal bovine serum (FBS, Thermo Fisher Scientific), penicillin (100 U/mL), and streptomycin (100 *μ*g/mL) at 37°C in an atmosphere of 5% CO_2_. Primary chondrocytes were trypsinized by 0.25% trypsin-EDTA (trypsin-ethylenediaminetetraacetic acid; Thermo Fisher Scientific) and replated. Chondrocytes at passage one were used for subsequent experiments.

### 2.2. *In Vitro* Culture of Human Chondrocytes and Treatments with IL-1*β* and Melatonin

#### 2.2.1. Chondrocyte Treatment

Cultured chondrocytes were maintained as a monolayer in DMEM/F12 with 10% FBS at 37°C. To establish an *in vitro* arthritic microenvironment, recombinant IL-1*β* at 5 ng/mL (Peprotech, Rocky Hill, NJ, USA) was supplemented in the medium for the indicated periods of time. Melatonin (Sigma-Aldrich, St. Louis, MO, USA) was dissolved in absolute ethanol (EtOH) at a stock concentration of 250 mM and then diluted in a complete medium at a concentration of 1 *μ*M or 100 *μ*M. Cells in the vehicle group were treated with an equal volume of EtOH (0.4 *μ*L per mL medium).

#### 2.2.2. Transfection of miR-140 Antagomir

miRNA-140 antagomir (antago miR-140) and negative control (antago miR-NC) oligonucleotides were obtained from GenePharma Co., Ltd. (Shanghai, China). The sequence of antago miR-140 is 5′-CUCCCUUCUCUUCUCCCGUCUU-3′ and NC is 5′-CUCCCUUCUCUUCUCCCGUCUU-3′. Chondrocytes were seeded in 6-well plates and transfected with miR-140 antagomir (100 nM) or NC miRNA using Lipofectamine 2000 (Thermo Fisher Scientific) according to the manufacturer's protocol. Forty-eight hours after transfection, the cells were treated with 5 ng/mL of IL-1*β* in the presence or absence of melatonin.

#### 2.2.3. Cell Proliferation

The detailed procedures are provided in the Supplementary data ([Supplementary-material supplementary-material-1]).

#### 2.2.4. Quantitative Real-Time Polymerase Chain Reaction (PCR) Analysis

Total RNA was extracted using the TRIzol® reagent (Thermo Fisher Scientific) according to the manufacturer's protocol. Complementary DNA (cDNA) was synthesized from 1 *μ*g of total RNA using the RevertAid First Strand cDNA Synthesis Kit (Thermo Fisher Scientific), and real-time PCR was performed with the iTap™ Universal SYBR® Green Supermix kit (Bio-Rad, Hercules, CA, USA) on a CFX96™ Real-Time PCR System (Bio-Rad). Transcript levels of *COL2A1* (type II collagen), *ACAN* (aggrecan), SOX9 (SRY-box-containing gene 9), *ADAMTS4*, *ADAMTS5*, *MMP9*, and *MMP13* were evaluated with *GAPDH* (glyceraldehyde-3-phosphate dehydrogenase) as an internal standard. Relative transcript levels of target genes were calculated using the comparative Ct (2^−*ΔΔ*Ct^) method. The primer sequences used in this study are listed in Supplementary [Supplementary-material supplementary-material-1].

#### 2.2.5. Immunofluorescence

The detailed procedures are provided in the Supplementary data.

#### 2.2.6. Western Blotting

The detailed procedures are provided in the Supplementary data.

### 2.3. *In Vivo* Experiments

#### 2.3.1. Mouse Model of Surgically Induced OA

Animal experiments were performed according to the Guidelines for Animal Experimentation of Soochow University and with the approval of the Ethics Committee of the First Affiliated Hospital of Soochow University. Male, nine-week-old C57BL/6J mice were purchased from the Animal Center of Soochow University. To establish the mouse model of OA, DMM surgery was performed to induce mild instability of the knee according to a previous study [15]. Briefly, mice were anesthetized using an inhalation anesthesia system (chamber filled with 2.0% isoflurane plus 30% oxygen, RWD Life Science, Shenzhen, China). The medial meniscotibial ligaments (MML) in the right knees were transected using microsurgical scissors to cause the instability of the medial meniscus. As controls, sham surgery was performed in the left knees, in which the capsular incision was made but the MML was left intact. All surgeries were performed by the same person (ZY).

#### 2.3.2. Intra-Articular Injection of Melatonin and the miRNA-140 Antagomir

Melatonin was dissolved in absolute ethanol and diluted in saline (0.9% NaCl) to yield a final concentration of 10 mg/mL. After surgery, the sham-op and DMM-op mice were treated with equal amounts (10 *μ*L) of melatonin or saline via intra-articular injection through the patellar tendon. The mice were injected twice a week for four weeks, then euthanized to collect samples of their medial femoral condyle cartilage. For miR-140 inhibition experiments, DMM-op mice were treated with equal amounts (10 *μ*L) of miRNA control (NC) or miR-140 antagomir (250 nM) via intra-articular injection on days 3 and 7 postsurgery along with melatonin administration.

#### 2.3.3. Histology and Immunohistochemistry

Dissected mouse knees were fixed in 10% formalin and decalcified in 10% EDTA (pH = 7.4, Sigma-Aldrich) for 2 weeks. After decalcification, each specimen was embedded in paraffin and sagittally sectioned at a thickness of 6 *μ*m. For histological analysis, the sections were stained with hematoxylin and eosin (H&E) and Safranin O (S.O.)/Fast Green (Sigma-Aldrich). Histological images were taken with a bright-field microscope (Zeiss Axiovert 200, Oberkochen, Germany). The slides stained with Safranin O/Fast Green were scored by three independent investigators (ZY, LT, and HF) blinded to group assignment, using the Osteoarthritis Research Society International (OARSI) scoring system [16].

For immunohistochemistry, the paraffin-embedded sections were dewaxed using xylene and hydrated in decreasing graded ethanol solutions. The slides were incubated with 1% hydrogen peroxide (H_2_O_2_; Sigma-Aldrich) for 30 min and then treated with 2 mg/mL testicular hyaluronidase (Sigma-Aldrich) for 30 min at 37°C. The slides were blocked in 1.5% goat serum, followed by incubation with specific anti-Collagen II (COL II; ab34712) or anti-Collagen I (COL I; ab34710, Abcam) primary antibodies overnight at 4°C. A secondary antibody of biotinylated goat anti-rabbit (Vector Laboratories, Burlingame, CA, USA) was applied for 30 min, after which avidin-biotin complex amplification (Vectastain ABC kit, Vector Laboratories) was used. Finally, immunohistochemistry was detected using 3,3′-diaminobenzidine (DAB; Vector Laboratories) as a substrate, and counterstaining was performed with hematoxylin. The percentage of COL II- or COL I-positive cells was counted (per 100 *μ*m^2^ area in each section) for quantitative evaluation.

### 2.4. Microarray

The detailed procedures are provided in the Supplementary data.

### 2.5. miRNA Expression Analysis

The detailed procedures are provided in the Supplementary data.

### 2.6. Statistical Analysis

All statistical analyses were performed using the SPSS 13.0 statistical software (SPSS Inc., Chicago, IL, USA). Data points are presented as means ± standard error of mean (S.E.M.) for at least three independent experiments. Statistical significance was determined using the two-tailed Student's *t*-test for comparisons between two groups and one-way Analysis of Variance (ANOVA) with Tukey's post hoc test for multiple group comparisons. *p* < 0.05 (∗) was considered statistically significant and *p* < 0.01 (∗∗) was considered highly significant.

## 3. Results

### 3.1. The Effect of Melatonin on Human Chondrocytes under Normal Culture Conditions

**Figure 1:**
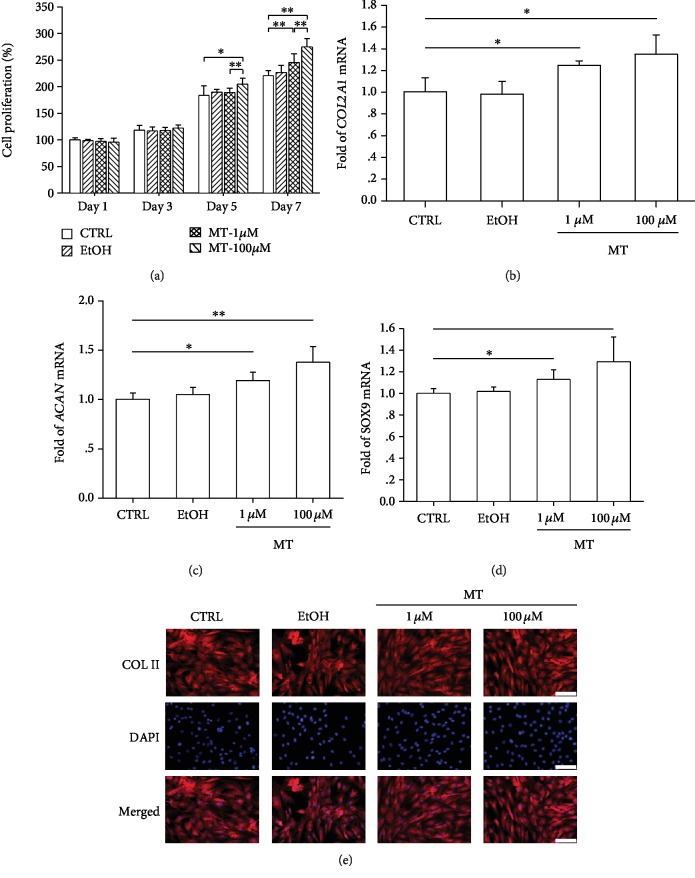
The effect of melatonin on cell proliferation and matrix synthesis in human articular chondrocytes. (a) Chondrocytes were treated with melatonin (MT) at concentrations of 1 *μ*M and 100 *μ*M, and cell proliferation was evaluated using the CCK-8 assay. Untreated cells served as the control group (CTRL) and cells treated with ethanol (EtOH) served as the vehicle group. (b–d) The mRNA levels of chondrogenic genes, including *COL2A1* (b), *ACAN* (c), and *SOX9* (d), were quantified with real-time PCR using *GAPDH* for normalization. (e) The protein expression of type II collagen was confirmed through immunofluorescence. Scale bar = 50 *μ*m. Values are the mean ± S.E.M. of six independent experiments (*n* = 6) in cell proliferation assays and four independent experiments (*n* = 4) in PCR experiments. Statistically significant differences are indicated by ∗ where *p* < 0.05 or ∗∗ where *p* < 0.01 between the indicated groups.

Human articular chondrocytes were treated with melatonin at 1 *μ*M and 100 *μ*M concentrations. The CCK-8 results showed that treatment with 100 *μ*M melatonin improved cell proliferation by 11.4% on day 5 and 24.5% on day 7 (Figure 1(a)). Furthermore, melatonin treatments significantly upregulated transcript levels of *COL2A1* by 24.0% at 1 *μ*M and 34.2% at 100 *μ*M (Figure 1(b)). Similarly, gene expressions of *ACAN* (Figure 1(c)) and *SOX9* (Figure 1(d)) were both enhanced by melatonin treatments. The immunofluorescence experiment confirmed the protein expression of Collagen II in the control and melatonin-treated chondrocytes (Figure 1(e)).

### 3.2. The Effect of Melatonin on Matrix Synthesis in IL-1*β*-Stimulated OA Chondrocytes

**Figure 2:**
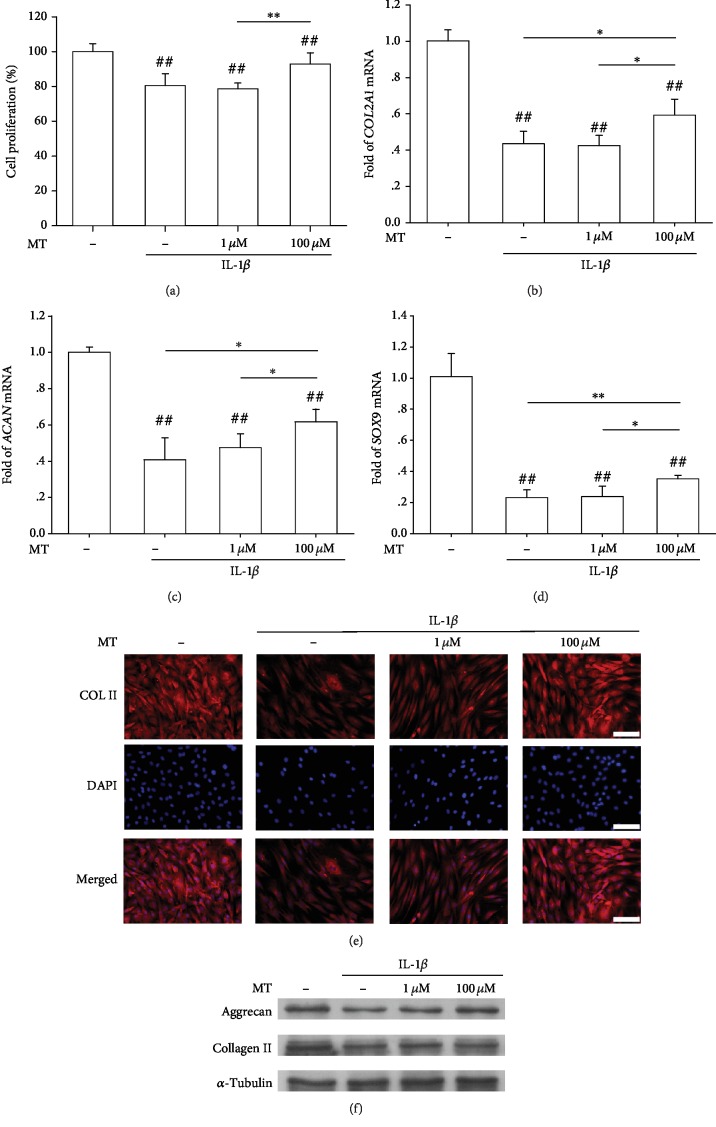
*In vitro* treatments with melatonin protected human articular chondrocytes from IL-1*β*-induced OA environment. (a) Chondrocytes were exposed to IL-1*β* (5 ng/mL) and then treated with melatonin (MT) at concentrations of 1 *μ*M and 100 *μ*M. Cell proliferation was evaluated using the CCK-8 assay. (b–d) The mRNA levels of chondrogenic genes, including *COL2A1* (b), *ACAN* (c), and *SOX9* (d), were quantified. (e) The protein expression of type II collagen was confirmed by an immunofluorescence experiment. Scale bar = 50 *μ*m. (f) The protein levels of aggrecan and type II collagen in melatonin-treated cells were determined using Western blot assays. Values are the mean ± S.E.M. of six independent experiments (*n* = 6) in cell proliferation assays, four independent experiments (*n* = 4) in PCR experiments, and three independent experiments (*n* = 3) in Western blot assays. Statistically significant differences are indicated by # where *p* < 0.05 or ## where *p* < 0.01 vs. the CTRL group and ∗ where *p* < 0.05 or ∗∗ where *p* < 0.01 between the indicated groups.

To investigate the protective effect of melatonin on OA chondrocytes, we first established an *in vitro* OA environment by adding 5 ng/mL of IL-1*β* during cell culturing. The CCK-8 assay showed that cell proliferation was reduced by 19.6% in the presence of IL-1*β*, whereas melatonin treatment (100 *μ*M) significantly increased cell proliferation by 15.4% compared with the IL-1*β* group (Figure 2(a)). Real-time PCR data suggested that, in IL-1*β*-stimulated chondrocytes, transcript levels of *COL2A1* were downregulated by 56.6%. Treatment with 100 *μ*M melatonin significantly upregulated the gene expression of *COL2A1* by 36.3% compared with the IL-1*β* group, but the level was still 40.8% lower than that of the control group (Figure 2(b)). Consistently, the high concentration of melatonin increased the transcript levels of *ACAN* by 51.4% (Figure 2(c)) and *SOX9* by 52.1% (Figure 2(d)) compared to the IL-1*β*-treated chondrocytes. The immunofluorescence staining showed that the synthesis of Collagen II in chondrocytes was suppressed by IL-1*β* treatment but restored by melatonin supplementation (Figure 2(e)). Western blot data confirmed that protein levels of cartilage ECM, such as Collagen II and aggrecan, were significantly increased by 100 *μ*M of melatonin (Figure 2(f) and Supplementary Figures [Supplementary-material supplementary-material-1] and [Supplementary-material supplementary-material-1]).

### 3.3. Inhibition of IL-1*β*-Induced Matrix Degradation by Melatonin in Chondrocytes

**Figure 3:**
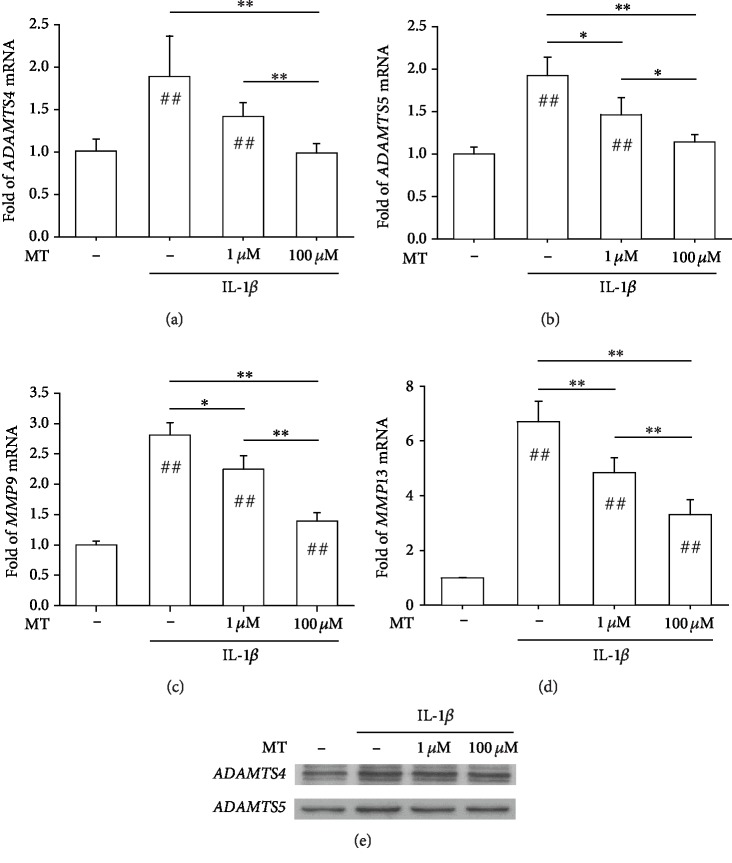
*In vitro* treatments with melatonin inhibited the expression of matrix-degrading enzymes stimulated by IL-1*β*. (a–d) The mRNA levels of matrix-degrading enzyme genes, including *ADAMTS4* (a), *ADAMTS5* (b), *MMP9* (c), and *MMP13* (d), were quantified with real-time PCR using *GAPDH* for normalization. (e) The protein levels of matrix-degrading enzymes in melatonin-treated cells were determined using Western blot assays. Values are the mean ± S.E.M. of four independent experiments (*n* = 4) in PCR experiments and three independent experiments (*n* = 3) in Western blot assays. Statistically significant differences are indicated by # where *p* < 0.05 or ## where *p* < 0.01 vs. the CTRL group and ∗ where *p* < 0.05 or ∗∗ where *p* < 0.01 between the indicated groups.

We further investigated the effect of melatonin on IL-1*β*-induced matrix-degrading enzymes. MMPs and ADAMTSs are important proteases that can degrade a wide range of matrix components. Real-time PCR data showed that IL-1*β* stimulation resulted in an 87.2% increase in the gene expression of *ADAMTS4*, whereas melatonin treatment downregulated its expression in a dose-dependent manner (by 24.9% at 1 *μ*M and by 47.8% at 100 *μ*M; Figure 3(a)). Similarly, treatment with 100 *μ*M melatonin significantly decreased the transcript levels of *ADAMTS5* by 40.6% (Figure 3(b)), *MMP9* by 50.5% (Figure 3(c)), and *MMP13* by 50.7% (Figure 3(d)). The inhibitory effects of melatonin on these matrix-degrading enzymes at the protein levels were confirmed by Western blot assays (Figure 3(e) and Supplementary Figures [Supplementary-material supplementary-material-1]–[Supplementary-material supplementary-material-1]).

### 3.4. Prevention of DMM-Induced OA in Mice by Intra-Articular Injection of Melatonin

**Figure 4:**
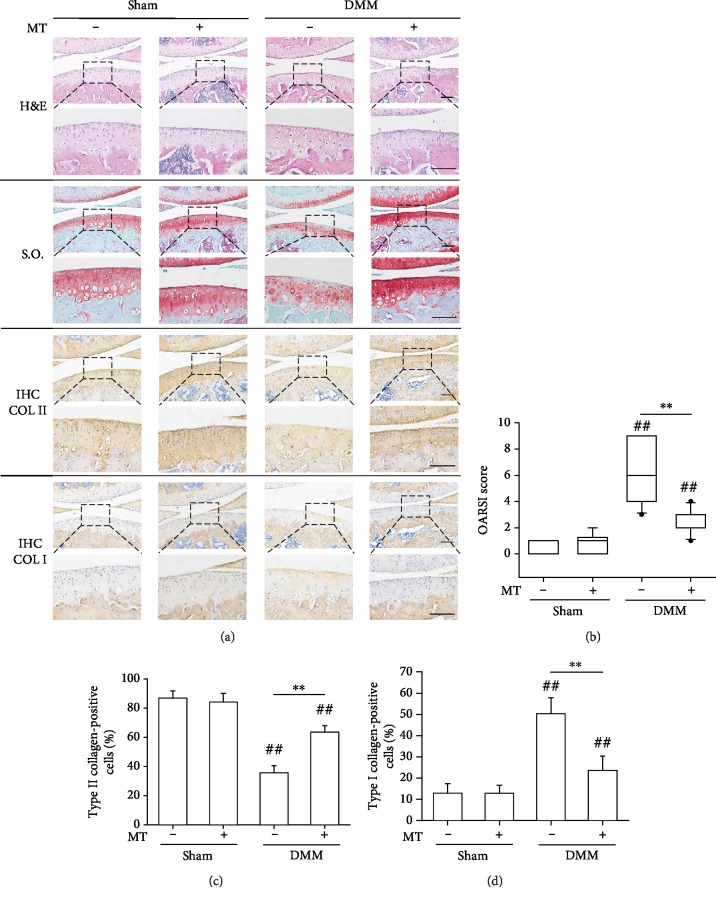
Intra-articular injection of melatonin prevented the progression of surgically induced OA in mice. DMM surgery was performed to induce OA, and after surgery, the sham-op and DMM-op mice were injected with equal amounts of melatonin or saline twice a week for four weeks. (a) Representative images of histological and immunohistochemical staining of the medial femoral condyle in OA mice. Sagittal sections of cartilage were stained by hematoxylin and eosin (H&E) and Safranin O (S.O.)/Fast Green. Immunohistochemical analyses were conducted to target COL II and COL I. Scale bar = 100 *μ*m. (b) OARSI scores were calculated based on the Safranin O/Fast Green staining results. (c, d) The percentages of COL II-positive (c) or COL I-positive (d) chondrocytes were counted. In each section, the quantitative analyses were counted at three random regions and then averaged. Values are the mean ± S.E.M. of ten independent experiments (*n* = 10). Statistically significant differences are indicated by ## where *p* < 0.01 vs. the CTRL group and ∗∗ where *p* < 0.01 between the indicated groups.

To investigate the antiarthritic effects of melatonin, sham-op and DMM-op mice were treated with melatonin via intra-articular injection. Histological and immunohistochemical analyses confirmed the loss of proteoglycan and Collagen II in the DMM group, whereas melatonin treatment prevented DMM-induced cartilage matrix destruction (Figure 4(a)). In particular, the positive staining of Collagen I was observed in the DMM-treated mice, which is a typical marker for OA development. In contrast, melatonin treatment suppressed the expression of Collagen I. In the melatonin-treated OA mice, the OARSI score was significantly reduced compared to that of the DMM group (Figure 4(b)). Consistently, the percentage of chondrocytes that stained positively for Collagen II was increased in the DMM+melatonin group (Figure 4(c)), while the percentage of Collagen I-positive cells was decreased (Figure 4(d)). These results indicate that intra-articular injection of melatonin successfully attenuated surgically induced OA progression in mice by modulating cartilage matrix homeostasis.

### 3.5. Melatonin-Mediated Differential miRNA Expression Profiles in Human Chondrocytes

**Figure 5:**
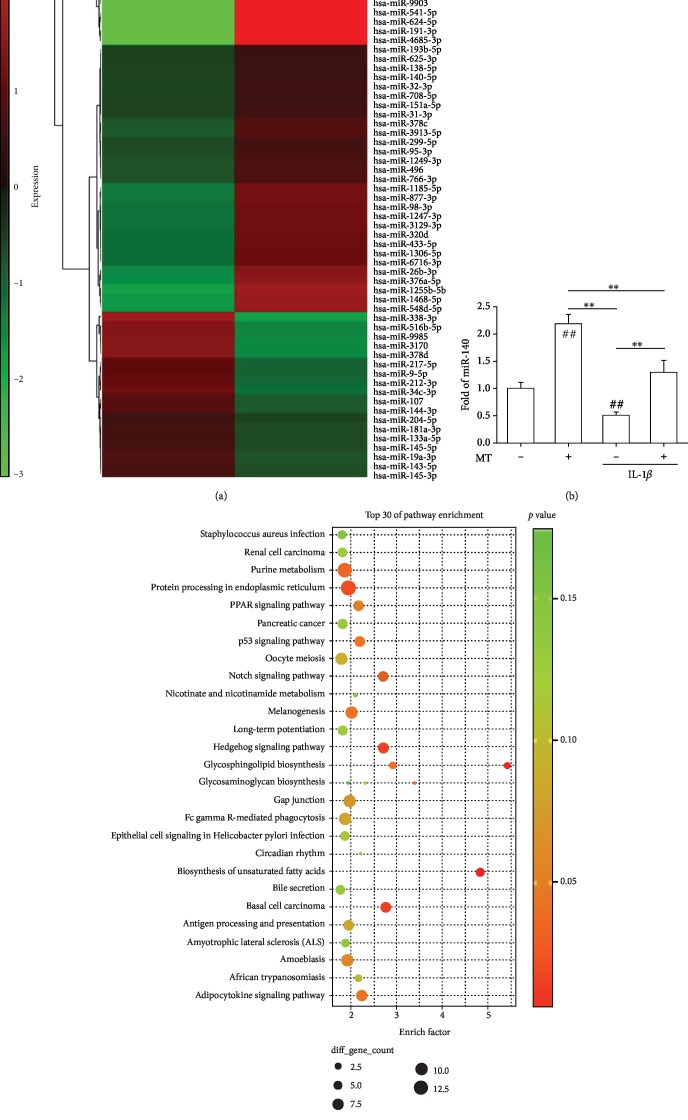
Identification of miRNAs that are differentially expressed in melatonin-treated human articular chondrocytes. (a) Differentially expressed miRNAs in chondrocytes in response to melatonin were illustrated as a heat map. The color bars on the left of the heat map indicate gene expression level; red denotes high expression and blue denotes low expression, relative to the median. (b) Real-time PCR validation of miR-140 expression in IL-1*β*-treated and melatonin-treated chondrocytes. Values are the mean ± S.E.M. of four independent experiments (*n* = 4) in PCR. (c) Pathway enrichment analysis for targets of miRNAs. The size of the dots (gene count) represents the number of genes (predicted targets of differentially expressed miRNAs) involved in a given biological process. The color of the dots represents *p* value. The terms are sorted alphabetically. Statistically significant differences are indicated by ## where *p* < 0.01 vs. the CTRL group and ∗∗ where *p* < 0.01 between the indicated groups.

To identify the differentially expressed miRNAs that were regulated by melatonin, microarray analysis was performed. As shown in Figure 5(a), after 72 h of stimulation of chondrocytes with 100 *μ*M melatonin, the heat map of miRNA expression patterns revealed that a total of 50 differentially expressed miRNAs (1.5-fold up- or downregulated) were identified (Supplementary [Supplementary-material supplementary-material-1]). Among them, 29 miRNAs were upregulated by melatonin-treated cells, including miR-193b-5p (3.00-fold), miR-26b-3p (2.86-fold), miR-140-5p (2.37-fold), and miR-95-3p (2.27-fold). On the other hand, 21 miRNAs were downregulated by melatonin treatment, including miR-9-5p (5.87-fold), miR-204-5p (4.05-fold), miR-145-5p (3.23-fold), and miR-181a-3p (2.29-fold). Considering the important role of miR-140 in the pathogenesis of OA, we performed real-time PCR assays to validate the changes of miR-140 expression. IL-1*β* stimulation resulted in a marked reduction in the level of miR-140 in human chondrocytes by 49.2%. Melatonin treatment upregulated the expression of miR-140 by 1.2-fold and 1.5-fold, respectively, compared with the CTRL and IL-1*β* groups (Figure 5(b)). To investigate specific miRNA-regulated gene pathways involved in the pathology of OA, pathway enrichment analysis was performed using DAVID. As shown in Figure 5(c), 27 pathways were significantly enriched, including protein processing in the endoplasmic reticulum, PPAR signaling pathway, p53 signaling pathway, Notch signaling pathway, and Hedgehog signaling pathway.

### 3.6. Reverse of Melatonin-Mediated Antiarthritic Effects by Inhibition of miR-140

**Figure 6:**
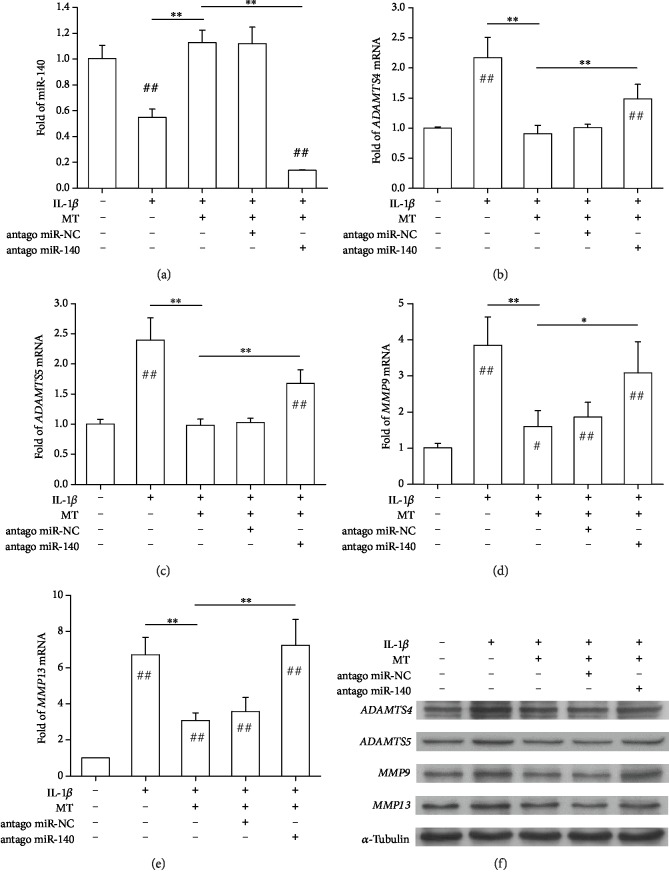
Inhibition of miR-140 intensified IL-1*β*-induced matrix degradation in human chondrocytes. Chondrocytes were treated with 5 ng/mL IL-1*β* and 100 *μ*M melatonin (MT). To inhibit miR-140 expression, chondrocytes were transfected with miRNA-140 antagomir (antago miR-140), and cells that were transfected with negative control miRNA (antago miR-NC) served as a control. (a) The expression of miR-140 was decreased in antagomir-treated chondrocytes. (b–e) The mRNA levels of matrix-degrading enzyme genes, including *ADAMTS4* (b), *ADAMTS5* (c), *MMP9* (d), and *MMP13* (e), were quantified with real-time PCR using *GAPDH* for normalization. (f) Protein levels of matrix-degrading enzymes in melatonin-treated cells were determined using Western blot assays. Values are the mean ± S.E.M. of four independent experiments (*n* = 4) in PCR experiments and three independent experiments (*n* = 3) in Western blot assays. Statistically significant differences are indicated by # where *p* < 0.05 or ## where *p* < 0.01 vs. the CTRL group and ∗ where *p* < 0.05 or ∗∗ where *p* < 0.01 between the indicated groups.

To investigate the role of miR-140 in melatonin-mediated antiarthritic effects, chondrocytes were transfected with miR-140 antagomir or an NC miRNA. Real-time PCR experiments confirmed that miR-140 antagomir transfection resulted in an 87.7% reduction in the level of miR-140, whereas miR-NC transfection barely affected miR-140 expression (Figure 6(a)). Inhibition of miR-140 reversed the inhibitory effects mediated by melatonin on matrix-degrading enzymes in IL-1*β*-stimulated chondrocytes. Compared to the IL-1*β*+MT group, miR-140 antagomir transfection increased the gene expression of *ADAMTS4* by 64.0% (Figure 6(b)), *ADAMTS5* by 70.8% (Figure 6(c)), *MMP9* by 93.1% (Figure 6(d)), and *MMP13* by 1.4-fold (Figure 6(e)). The protein levels of matrix-degrading enzymes were confirmed by Western blot experiments (Figure 6(f) and Supplementary [Supplementary-material supplementary-material-1]).

**Figure 7:**
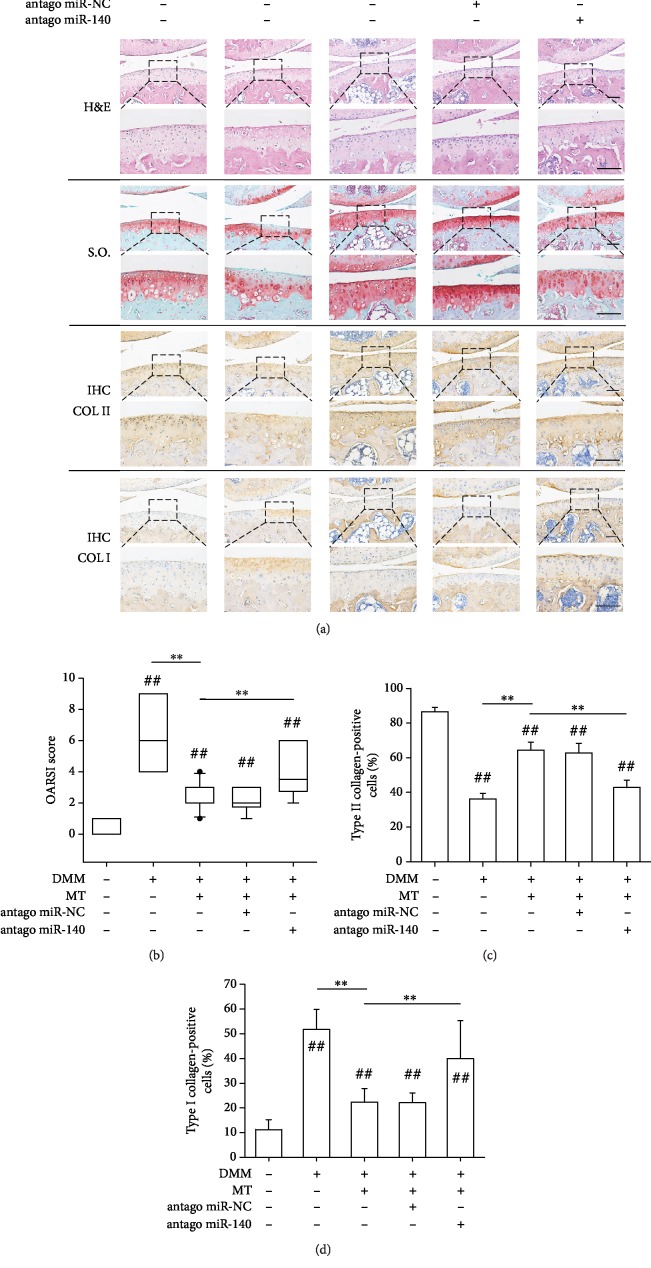
Inhibition of miR-140 counteracted the antiarthritic effects of melatonin in surgically induced OA mice. After the destabilization of the medial meniscus (DMM) surgery, the sham-op and DMM-op mice were injected with melatonin (MT), negative control miRNA (antago miR-NC), or miRNA-140 antagomir (antago miR-140). (a) Representative images of histological (H&E and Safranin O/Fast Green) and immunohistochemical (COL II and COL I) staining of the medial femoral condyle in OA mice. Scale bar = 100 *μ*m. (b) OARSI scores were calculated based on the Safranin O/Fast Green staining results. (c, d) The percentages of COL II-positive (c) or COL I-positive (d) chondrocytes were counted. In each section, the quantitative analyses were counted at three random regions and then averaged. Values are the mean ± S.E.M. of ten independent experiments (*n* = 10). Statistically significant differences are indicated by ## where *p* < 0.01 vs. the CTRL group and ∗∗ where *p* < 0.01 between the indicated groups.

We further investigated the effect of intra-articular injection of miR-140 antagomir on OA progression. After the DMM surgery, OA mice were injected with miR-140 antagomir along with melatonin. Histology results showed reduced levels of proteoglycan and Collagen II in the antagomir-treated mice (Figure 7(a)). Importantly, immunohistochemical analysis revealed strong positive staining for Collagen I in the antagomir-treated mice. In the miR-140 antagomir group, the OARSI score was significantly increased (Figure 7(b)) and the percentage of Collagen II-positive chondrocytes decreased (Figure 7(c)), in contrast to the increased percentage of Collagen I-positive cells (Figure 7(d)). These results suggest that inhibition of miR-140 completely abrogated the antiarthritic effect of melatonin.

## 4. Discussion

The imbalance between the anabolism and catabolism of the cartilage matrix is the hallmark of OA, which is induced by the increased number of proinflammatory cytokines. IL-1*β* is an important mediator of joint inflammation; not only does it blunt the synthesis of cartilage ECM, it also stimulates the expression of matrix-degrading enzymes. It was reported that IL-1*β* inhibited chondrogenesis of human MSCs through activation of the nuclear factor *κ*-light-chain enhancer of activated B cell (NF-*κ*B) signaling pathway [17]. In this study, we used IL-1*β* to mimic the OA inflammatory environment *in vitro* and showed that IL-1*β* stimulation significantly suppressed the synthesis of cartilage-specific ECM (e.g., Collagen II and aggrecan). However, treatment with melatonin promoted the expression of cartilage ECM at both the mRNA and protein levels, even in the presence of IL-1*β*. SOX9 plays an important role in chondrogenesis by regulating matrix protein synthesis during neocartilage formation [18]. Consistent with previous studies, we found that melatonin significantly increased the level of transcriptional factor SOX9 even in IL-1*β*-treated chondrocytes. In addition, chondrocyte proliferation was improved by melatonin treatments in a dose-dependent manner, in which SOX9 may contribute to an increased number of proliferating chondrocytes [19].

On the other hand, melatonin was demonstrated to have an inhibitory effect on cartilage ECM degradation. In this study, we demonstrated that, in IL-1*β*-stimulated chondrocytes, melatonin treatments led to a significant reduction in the expression of matrix-degrading enzymes such as ADAMTS4 and ADAMTS5. These two proteinases are responsible for aggrecan degradation during OA development, which are found at increased mRNA expression and protein levels in OA cartilage [20]. Knockout of *ADAMTS4* and *ADAMTS5* in mice protected the hyaline cartilage against proteoglycan degradation and decreased the severity of murine OA [21]. Inhibition of ADAMTS4 and ADAMTS5 by melatonin preserved the intact proteoglycan network in cartilage ECM, which may further protect the cartilage collagen network (especially Collagen II) from degradation induced by MMPs during OA progression [22]. In addition, we showed that melatonin significantly decreased MMP13 levels in IL-1*β*-induced chondrocytes, which may contribute to the attenuation of matrix loss in OA cartilage. However, the regulatory effect of melatonin on MMP13 is still disputable. Hong et al. suggested that melatonin promotes ECM remodeling in OA cartilage through the upregulation of MMP13 [23]. The different effects of melatonin on MMP13 may be due to the low dosage of melatonin and the complex interventions involved, which combined treadmill exercise with melatonin treatments of only up to 1 *μ*M.

In addition to the transcriptional regulations, the present study is the first to demonstrate that melatonin prevents OA-induced cartilage destruction by regulating the expression of matrix-degrading enzymes at the posttranscriptional level. Previous studies have reported that several miRNAs are involved in the pathogenesis of OA, such as miR-9, miR-27b, miR-34a, miR-140, and miR-146 [24]; however, the effects of melatonin on modulating miRNAs in OA chondrocytes have not been reported. In this study, we first identified miR-140 as a melatonin-responsive miRNA in chondrocytes, characterized by a significant upregulation by melatonin with or without IL-1*β* stimulation. miR-140, a cartilage-specific miRNA, has been shown to inhibit the NF-*κ*B signaling pathway in IL-1*β*-treated articular chondrocytes and to reduce cartilage ECM breakdown via downregulation of ADAMTS5 [25]. The negative effect of miR-140 on MMP9 has been reported to inhibit breast cancer invasion [26], while estrogen has been shown to suppress MMP13 expression through the upregulation of miR-140 in the development of menopausal arthritis [27]. In addition, the microarray results in this study demonstrated that melatonin modulated the expression of several miRNAs involved in cartilage development and OA progression. For example, we observed that melatonin treatment upregulated the expression of miR-95 and downregulated the expression of miR-9 and miR-204. miR-95 has been reported to promote cartilage matrix expression by directly inhibiting histone deacetylase 2/8 (HDAC2/8) [28]. Previous studies have demonstrated that miR-9 [29] and miR-204 [30] markedly increase in OA cartilage and contribute to the disrupted matrix homeostasis via upregulation of IL-6 expression and inhibition of the sulfated proteoglycan biosynthesis pathway, respectively. Therefore, further studies are necessary to investigate the effects of melatonin on other OA-related miRNAs and to unravel the regulation of melatonin-responsive miRNAs on cartilage matrix homeostasis.

The underlying mechanisms by which melatonin regulates miR-140 expression are not fully understood. The miR-140 gene was reported to be located in an intronic region of its host gene, the WW domain-containing E3 ubiquitin protein ligase 2 (WWP2) [31]. Yang et al. confirmed that miR-140 was cotranscribed with the C-terminal isoform of WWP2 and was directly induced by SOX9 via binding to the WWP2 gene. miR-140 was crucial for chondrocyte proliferation through its targeting of Sp1, which is the activator of p15^INK4b^. This was because the inhibition of miR-140 resulted in the arrest of proliferation in micromass cultures [32]. In addition, Tardif et al. demonstrated that regulation of miR-140 expression can be independent of WWP2. In normal chondrocytes, mechanotransduction signals induced the translocation of the nuclear factor of activated T-cells 3 (NFAT3) to the nucleus and subsequently activated the expression of miR-140, whereas in OA chondrocytes, overexpression of transforming growth factor-*β* (TGF-*β*) resulted in the phosphorylation of mothers against decapentaplegic homolog 3 (SMAD3) that directly inhibited miR-140 [33]. Moreover, a previous study reported that in OA chondrocytes, the increased levels of miR-145 directly inhibited miR-140 expression, resulting in a reduction in cartilage ECM gene expression and an increase in the ECM-degrading enzyme MMP13 [34]. In this study, we found that melatonin suppressed the expression of miR-145 in human chondrocytes, suggesting that the attenuation of OA progression by melatonin may involve other miRNAs. Therefore, the melatonin-mediated regulation of cartilage ECM synthesis and degradation at the posttranscriptional level will be an important area for future investigation. Furthermore, based on the pathway enrichment analysis, we found that several miRNA-regulated gene pathways were involved in the melatonin-mediated antiarthritic effects, such as the PPAR, p53, and Notch signaling pathways. For example, the Notch signaling pathway regulates cell differentiation and apoptosis through a single-pass transmembrane cell surface receptor [35] and has been reported to play an important role in articular cartilage degradation and OA development [36]. Li et al. showed that treatment with melatonin activated the Notch signaling pathway and increased the proliferation and differentiation of neural stem cells via downregulation of miR-363 [37]. However, the underlying mechanisms of melatonin-mediated signaling pathways including the Notch in OA chondrocytes will be investigated in our future studies.

The delivery of melatonin in animal studies is traditionally through oral administration or intraperitoneal injection. Daily oral administration of melatonin (100 mg/kg body weight/day) has been reported to prevent ovariectomy-induced bone degeneration by increasing bone formation in mice [38], while intraperitoneal melatonin injections were proven to effectively alleviate titanium particle-induced inflammatory osteolysis in a murine calvarial model [39]. However, considering that chondrocytes uptake nutrients mainly from the synovial fluid, intra-articular injection is an effective way to administer melatonin into the articular cavity in DMM-induced OA mice. Intra-articular injection of melatonin successfully ameliorated OA progression by preserving cartilage ECM homeostasis through miR-140. The preventive effects of melatonin on OA were confirmed by suppressing miR-140-mediated ECM destruction, since inhibition of miR-140 by the antagomir transfection completely neutralized the antiarthritic effects of melatonin in DMM-operated mice. In agreement with our results, Si et al. showed that overexpression of miR-140 by intra-articular injection inhibited the expression of matrix-degrading enzymes and protected cartilage from surgically induced OA in a rat model [40].

One limitation of this study is that we investigated the therapeutic effects of melatonin on the early stage of OA; it is unknown whether intra-articular injection of melatonin will show the same protective effects on late-stage or other types of OA. Lim et al. showed that intra-articular injection of melatonin protected chondrocytes from oxidative stress-induced cartilage degradation through activation of the silent information regulator type 1 (SIRT1) signaling pathway [41]. Our future studies will seek to investigate the effects of melatonin-mediated miR-140 expression on late-stage OA.

## 5. Conclusions

In summary, we demonstrated that intra-articular injection of melatonin ameliorated the progression of OA by protecting cartilage from matrix destruction in surgically induced OA mice. In an *in vitro* IL-1*β*-induced arthritic environment, melatonin treatment enhanced the synthesis of matrix proteins and suppressed the expression of matrix-degrading enzymes in human articular chondrocytes. Further molecular experiments identified miR-140 as a melatonin-responsive miRNA and revealed that melatonin regulated the expression of proteinases at the posttranscriptional level by upregulating miR-140; it was evidenced by the fact that inhibition of miR-140 counteracts the antiarthritic effects of melatonin on OA-induced cartilage loss. Future studies are necessary to unveil the specific mechanisms by which melatonin modulates miR-140 expression and to investigate the therapeutic effects of melatonin on late-stage OA.

## Data Availability

The data used to support the findings of this study are available from the corresponding authors upon request.

## Supplementary Materials

Supplementary Materials Supplementary Figure 1: quantification of the protein levels of cartilage matrix components and matrix-degrading enzymes in melatonin-treated chondrocytes. Supplementary Figure 2: quantification of the protein levels of matrix-degrading enzymes in antago miR-140-treated chondrocytes. Supplementary Table 1: Primers used for real-time PCR. Supplementary Table 2: microRNA expression profiles of human articular chondrocytes in response to melatonin treatment. Supplementary experimental procedures: the protocols of cell proliferation, immunofluorescence, Western blotting, immunohistochemistry, microarray, and miRNA expression analysis.
