# Supplementary material for: Melatonin Prevents Osteoarthritis-Induced Cartilage Degradation via Targeting MicroRNA-140
Source: Oxid Med Cell Longev. 2019 Dec 14;2019:9705929. doi: 10.1155/2019/9705929 (PMC6935446; doi:10.1155/2019/9705929)
Supplement: Supplementary Materials — Supplementary Figure 1: quantification of the protein levels of cartilage matrix components and matrix-degrading enzymes in melatonin-treated chondrocytes. Supplementary Figure 2: quantification of the protein levels of matrix-degrading enzymes in antago miR-140-treated chondrocytes. Supplementary Table 1: Primers used for real-time PCR. Supplementary Table 2: microRNA expression profiles of human articular chondrocytes in response to melatonin treatment. Supplementary experimental procedures: the protocols of cell proliferation, immunofluorescence, Western blotting, immunohistochemistry, microarray, and miRNA expression analysis. [file 9705929.f1.docx]

**Supplementary Figure and Figure legends**


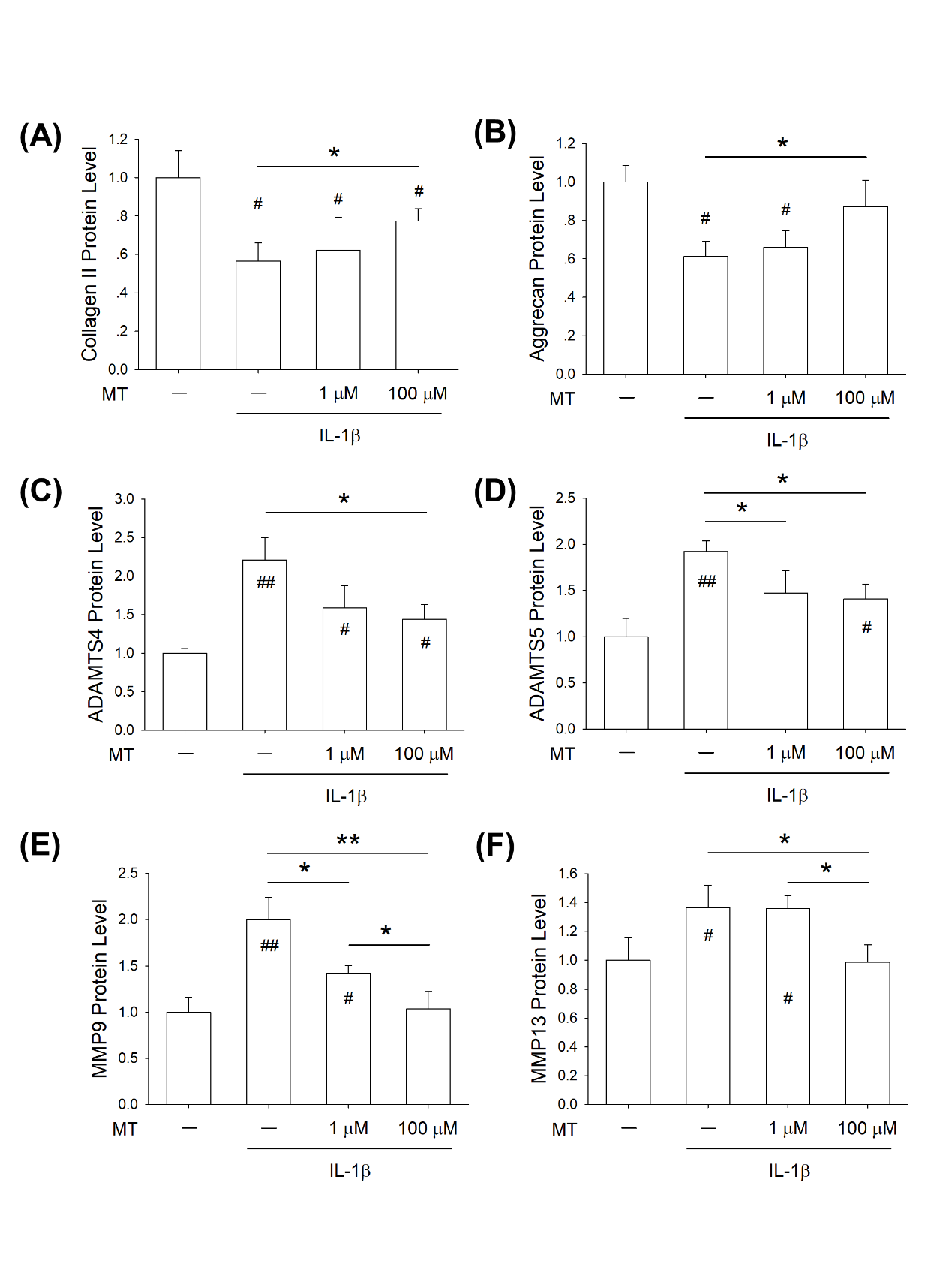


**Supplementary Figure 1.** The effects of melatonin treatment on the protein levels of cartilage matrix components and matrix-degrading enzymes in human articular chondrocytes. A-E. The protein levels of Collagen II (A), Aggrecan (B), ADAMTS4 (C), ADAMTS5 (D), MMP9 (E), and MMP13 (F) were quantified. Values are the mean ± S.E.M. of three independent experiments (*n* = 3) in Western blot assays. Statistically significant differences are indicated by ^#^ where p < 0.05 or ^##^ where p < 0.01 vs. the CTRL group and * where *p* < 0.05 or ** where *p* < 0.01 between the indicated groups.


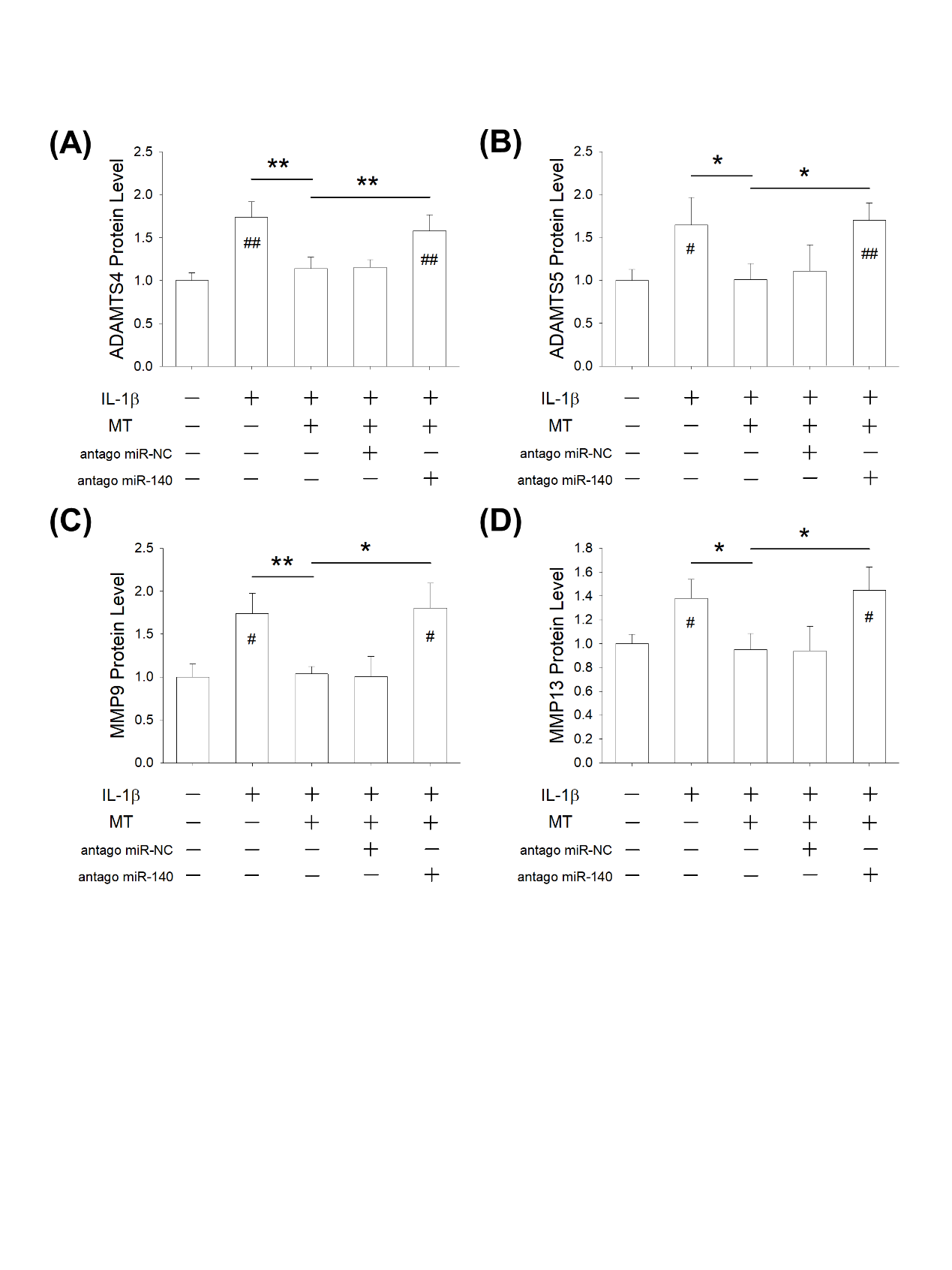


**Supplementary Figure 2.** Inhibition of miR-140 increased the protein levels of matrix-degrading enzymes in melatonin-treated chondrocytes. A-E. The protein levels of ADAMTS4 (A), ADAMTS5 (B), MMP9 (C), and MMP13 (D) were quantified. Values are the mean ± S.E.M. of three independent experiments (*n* = 3) in Western blot assays. Statistically significant differences are indicated by ^#^ where p < 0.05 or ^##^ where p < 0.01 vs. the CTRL group and * where *p* < 0.05 or ** where *p* < 0.01 between the indicated groups.

**Supplementary Table 1.** Primers used for real-time PCR

| **Gene** | **Forward Primer sequence(5’-3’)** | **Reverse Primer sequence(5’-3’)** |
| --- | --- | --- |
| *COL2A1* | TGGACGCCATGAAGGTTTTCT | TGGGAGCCAGATTGTCATCTC |
| *ACAN*  *SOX9* | ACTCTGGGTTTTCGTGACTCT  AGCGAACGCACATCAAGAC | ACACTCAGCGAGTTGTCATGG  CTGTAGGCGATCTGTTGGGG |
| *ADAMTS4* | CTGGCACCTACCTGACTGG | GTAACACGCCTAACAGGGCT |
| *ADAMTS5* | ACTACGATGCAGCTATCCTGT | GTCCCAACGTCTGCCATTC |
| *MMP9* | AGACCTGGGCAGATTCCAAAC | CGGCAAGTCTTCCGAGTAGT |
| *MMP13* | ACTGAGAGGCTCCGAGAAATG | GAACCCCGCATCTTGGCTT |
| *GAPDH* | AGAAAAACCTGCCAAATATGATGAC | TGGGTGTCGCTGTTGAAGTC |

**Supplementary Table 2.** MicroRNA expression profiles of human articular chondrocytes in response to melatonin treatment

| *No* | *microRNA* | *Fold Change* | *Regulation* |
| --- | --- | --- | --- |
| 1 | miR-1268b | 11.00 | Up |
| 2 | miR-541-5p | 10.08 | Up |
| 3 | miR-624-5p | 10.08 | Up |
| 4 | miR-4685-3p | 9.78 | Up |
| 5 | miR-191-3p | 9.58 | Up |
| 6 | miR-155-3p | 9.34 | Up |
| 7 | miR-3126-3p | 9.33 | Up |
| 8 | miR-202-5p | 9.08 | Up |
| 9 | miR-9903 | 9.07 | Up |
| 10 | miR-1255b-5p | 3.44 | Up |
| 11 | miR-1468-5p | 3.27 | Up |
| 12 | miR-548d-5p | 3.27 | Up |
| 13 | miR-376a-5p | 3.01 | Up |
| 14 | miR-193b-5p | 3.00 | Up |
| 15 | miR-26b-3p | 2.86 | Up |
| 16 | miR-1185-5p | 2.44 | Up |
| 17 | miR-877-3p | 2.44 | Up |
| 18 | miR-140-5p | 2.37 | Up |
| 19 | miR-1247-3p | 2.27 | Up |
| 20 | miR-3129-3p | 2.27 | Up |
| 21 | miR-95-3p | 2.27 | Up |
| 22 | miR-98-3p | 2.27 | Up |
| 23 | miR-320d | 2.19 | Up |
| 24 | miR-433-5p | 2.18 | Up |
| 25 | miR-6716-3p | 2.15 | Up |
| 26 | miR-1306-5p | 2.12 | Up |
| 27 | miR-31-3p | 2.13 | Up |
| 28 | miR-3913-5p | 1.65 | Up |
| 29 | miR-378c | 1.57 | Up |
| 30 | miR-107 | -1.62 | Down |
| 31 | miR-144-3p | -1.67 | Down |
| 32 | miR-217-5p | -1.89 | Down |
| 33 | miR-212-3p | -2.05 | Down |
| 34 | miR-34c-3p | -2.19 | Down |
| 35 | miR-516b-5p | -2.73 | Down |
| 36 | miR-181a-3p | -2.29 | Down |
| 37 | miR-9985 | -2.83 | Down |
| 38 | miR-378d | -2.89 | Down |
| 39 | miR-3170 | -2.90 | Down |
| 40 | miR-145-5p | -3.23 | Down |
| 41 | miR-143-5p | -3.36 | Down |
| 42 | miR-338-3p | -3.43 | Down |
| 43 | miR-204-5p | -4.05 | Down |
| 44 | miR-9-5p | -5.87 | Down |
| 45 | miR-1277-3p | -8.80 | Down |
| 46 | miR-4781-3p | -9.07 | Down |
| 47 | miR-874-3p | -9.07 | Down |
| 48 | miR-548ay-5p | -9.28 | Down |
| 49 | miR-6843-3p | -9.29 | Down |
| 50 | miR-5699-3p | -9.80 | Down |

**Experimental procedures**

**Cell proliferation**

The Cell Counting Kit-8 (CCK-8; Beyotime Institute of Biotechnology, Haimen, China) was used to assess cell proliferation. Chondrocytes were seeded into a 96-well plate at an initial density of 1×10^3^ cells per well. The following day, the cells were treated with melatonin at a final concentration of 1 μM or 100 μM for 1, 3, 5, 7 days. Cell proliferation was determined by CCK-8 following the manufacturer’s instructions. The absorbance at a wavelength of 450 nm was measured using a PowerWave XS spectrophotometer (BioTek, Winooski, VT, USA).

**Immunofluorescence**

Chondrocytes were fixed in 4% paraformaldehyde (Sigma-Aldrich) for 15 min and then incubated with 0.1 % Triton X-100 (Sigma-Aldrich) for permeabilization. Blocking was performed for 30 min at room temperature using 1% normal bovine serum, followed by incubation with a diluted primary antibody of Collagen II (ab34712, Abcam, Cambridge, MA, USA) for 60 min. The cells were further incubated with a secondary antibody corresponding to Alexa Fluor® 647-conjugated donkey anti-rabbit IgG (Abcam) for 60 min at room temperature, and the cell nuclei were counterstained with 4',6-diamidino-2-phenylindole (DAPI; Thermo Fisher Scientific). The images were observed using a Zeiss Axiovert 40CFL microscope (Zeiss, Oberkochen, Germany).

**Western blotting**

Total protein was extracted using RIPA lysis buffer (Beyotime) with a protease inhibitor cocktail (Thermo Fisher Scientific) and protein concentrations were determined using the BCA Protein Assay Kit (Beyotime). Equivalent amounts of protein lysate were separated using 10% sodium dodecyl sulfate-polyarylamide gel electrophoresis (SDS-PAGE) and transferred to nitrocellulose membranes (Beyotime). The membranes were blocked with blocking buffer (Beyotime) for 30 min and incubated overnight at 4°C with anti-Collagen II (ab34712), anti-aggrecan (ab3778), anti-ADAMTS4 (ab185722), anti-ADAMTS5 (ab41037), anti-MMP9 (ab38898), anti-MMP13 (ab39012), or anti-α-tubulin (ab7291, Abcam) primary antibodies. Blots were then incubated with horseradish peroxidase-conjugated secondary antibodies for 60 min at room temperature and visualized using an enhanced chemiluminescence solution (SuperSignal West Pico Substrate, Thermo Fisher Scientific) as recommended by the manufacturer. Gray values of the bands in scanned images were quantitatively evaluated using ImageJ software (National Institutes of Health, Bethesda, MD, USA) and was normalized to that of α-tubulin before comparison.

**Immunohistochemistry**

For immunohistochemistry, the paraffin-embedded sections were dewaxed using xylene and hydrated in decreasing graded ethanol solutions. The slides were incubated with 1% hydrogen peroxide (H_2_O_2_; Sigma-Aldrich) for 30 min and then treated with 2 mg/mL testicular hyaluronidase (Sigma-Aldrich) for 30 min at 37°C. The slides were blocked in 1.5% goat serum, followed by incubation with specific anti-collagen II (COL II; ab34712) or anti-collagen I (COL I; ab34710, Abcam) primary antibodies overnight at 4°C. A secondary antibody of biotinylated goat anti-rabbit (Vector Laboratories, Burlingame, CA, USA) was applied for 30 min, after which avidin-biotin complex amplification (Vectastain ABC kit, Vector Laboratories) was used. Finally, immunohistochemistry was detected using 3,3'-diaminobenzidine (DAB; Vector Laboratories) as a substrate and counterstaining was performed with hemotoxylin. The percentage of COL II- or COL I-positive cells was counted (per 100 μm^2^ area in each section) for quantitative evaluation.

**Microarray**

The miRNA expression profiles of human articular chondrocytes treated with or without melatonin (100 μM) were determined using the Affymetrix Human HTA2.0 expression microarrays (Affymetrix, Santa Clara, CA, USA). Total RNA was extracted using TRIzol^®^ reagent and quantified by the NanoDrop ND-2000 (Thermo Fisher Scientific). The RNA integrity was assessed using the Agilent Bioanalyzer 2100 (Agilent Technologies, Santa Clara, CA, USA). The microarray experiments were performed at Shanghai OE Biotech. Co., Ltd. (Shanghai, China) and data were analyzed using the Significant Analysis of Microarray software (SAM). The differentially expressed miRNAs were identified by a fold-value change of ≥ 1.5 in the SAM output results. Pathway enrichment analysis was performed using the online tool, the Database for Annotation, Visualization and Integrated Discovery (DAVID, http://david.abcc.ncifc rf.gov/). In this study, Gene Ontology (GO) functional annotation for biological process analysis was performed for predicted target genes of potential miRNAs.

**MiRNA expression analysis**

Total RNA containing miRNAs was isolated using the TRIzol^®^ reagent and the miRNAs were purified using the mirVana^TM^ miRNA Isolation Kit (Thermo Fisher Scientific) according to the manufacturer's instructions. The RNA was then reverse transcribed using the TaqMan^TM^ Advanced miRNA cDNA Synthesis Kit (Thermo Fisher Scientific). Human miR-140 and RNU6-6P (RNA, U6 small nuclear 6) expressions were evaluated using the TaqMan^TM^ Fast Advanced Master Mix (Thermo Fisher Scientific) on a CFX96^TM^ Real-Time PCR System. The primers used for miR-140 (4427975) and RNU6-6P (A25576) were purchased from Thermo Fisher Scientific.
